# The Dual Regulation of Apoptosis by Flavivirus

**DOI:** 10.3389/fmicb.2021.654494

**Published:** 2021-03-24

**Authors:** Yuhong Pan, Anchun Cheng, Mingshu Wang, Zhongqiong Yin, Renyong Jia

**Affiliations:** ^1^Research Center of Avian Disease, College of Veterinary Medicine, Sichuan Agricultural University, Chengdu, China; ^2^Institute of Preventive Veterinary Medicine, Sichuan Agricultural University, Chengdu, China; ^3^Key Laboratory of Animal Disease and Human Health of Sichuan Province, Chengdu, China

**Keywords:** apoptosis, flavivirus, Dengue virus, Japanese encephalitis virus, West Nile virus, Zika virus

## Abstract

Apoptosis is a form of programmed cell death, which maintains cellular homeostasis by eliminating pathogen-infected cells. It contains three signaling pathways: death receptor pathway, mitochondria-mediated pathway, and endoplasmic reticulum pathway. Its importance in host defenses is highlighted by the observation that many viruses evade, hinder or destroy apoptosis, thereby weakening the host’s immune response. Flaviviruses such as Dengue virus, Japanese encephalitis virus, and West Nile virus utilize various strategies to activate or inhibit cell apoptosis. This article reviews the research progress of apoptosis mechanism during flaviviruses infection, including flaviviruses proteins and subgenomic flaviviral RNA to regulate apoptosis by interacting with host proteins, as well as various signaling pathways involved in flaviviruses-induced apoptosis, which provides a scientific basis for understanding the pathogenesis of flaviviruses and helps in developing an effective antiviral therapy.

## Flavivirus

Flavivirus belong to the Flaviviridae family and have 70 different antigen-related members. Flavivirus is an emerging arthropod-borne virus that causes huge global health problems. According to the epidemic reports, Dengue virus (DENV), Japanese encephalitis virus (JEV), West Nile virus (WNV), Zika virus (ZIKV), Yellow fever virus (YFV), and tick-borne Encephalitis virus (TBEV) are major human pathogenic flaviviruses (Rastogi et al., 2016). They are responsible for the illness ranging from mild flu symptoms to severe hemorrhagic, neurological and cognitive manifestations that cause death.

### Structure

**FIGURE 1:**
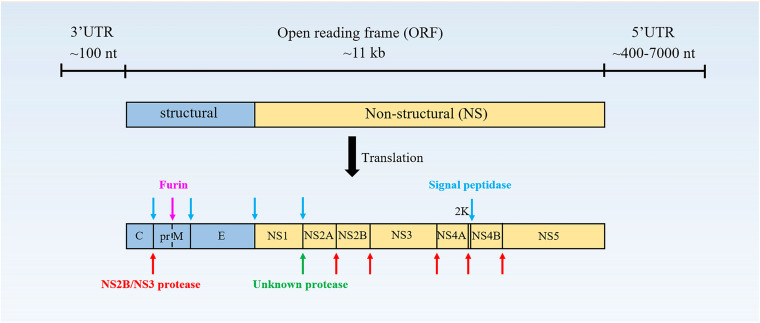
The structure of the flavivirus genome. The protein is cleaved co- and post-translationally to form capsid (C), membrane (M), and envelope (E) structural proteins and non-structural proteins (NS)-1, 2A, 2B, 3, 4A, 4B, and 5 (Vicenzi et al., 2018).

Flavivirus is a single-stranded, positive-polarity RNA virus with a genome of approximately 11 kb, which has only one open reading frame (ORF) flanked by a 5′-untranslated region (UTR) and a 3′-UTR (Figure 1). The ORF encodes a polyprotein of ∼3400 aa residues, which is cleaved by viral and host protease to produce three structural proteins [capsid (C); precursor of M (prM), and envelope (E)] and seven non-structural (NS) proteins (NS1, NS2A/2B, NS3, NS4A/4B, and NS5) (Tao et al., 2012; Wanjun et al., 2012; Huang et al., 2018).

### Life Cycle

**FIGURE 2:**
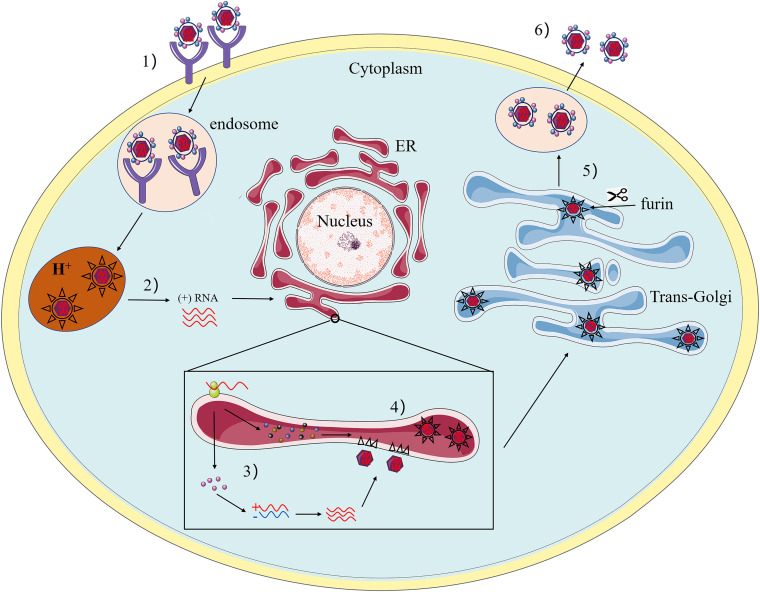
The Flavivirus life cycle. **(1)** The viruses attach to the surface of a host cell and internalize by receptor-mediated endocytosis. **(2)** Acidification of the endosomal vesicle triggers conformational changes in the virion, fusion of the viral envelope and cell membranes, releasing the viral genome into the cytoplasm. **(3)** The positive-sense RNA is translated into a single polyprotein that is processed co- and post-translationally by viral and host proteases. The viral RNA-dependent RNA polymerase replicates the viral genome in specialized ER-derived membrane compartments. **(4)** Virus assembly occurs on the surface of the ER, the immature viral particles bearing prM and E bud into ER rumen after nucleocapsid formation with viral RNA. The resultant non-infectious, immature viral are transported to the trans-Golgi network (TGN). **(5)** The host protease furin cleaved of prM to M, generating mature infectious particles. **(6)** Mature virions are subsequently released by exocytosis.

Flaviviruses share a common mechanism of propagation in host cells. Initially, they enter target cells via receptor-mediated endocytosis and transfer to endosomes. The acidic environment in the endosomal lumen triggers conformational changes of the glycoprotein on the surface of the virus, causing the virus envelope to fuse with the endosomal membranes (Bressanelli et al., 2004). Subsequently, the disintegration of the viral capsid (“uncoating”) delivers the RNA genome to the cytoplasm, completing the entry process. The positive polarity genomes then serve as templates for translation and replication. When the viral RNA is translated into a precursor protein at the endoplasmic reticulum (ER), this polypeptide is coordinated and post-translationally processed by the viral and host protease to produce three structural proteins and seven non-structural proteins. After translation of input genomic RNA, the RNA-dependent RNA polymerase (RdRp) NS5 copies complementary negative-stand RNA from genomic RNA, which serves as a template for the synthesis of new positive-strand viral RNA (Brinton, 2002). Immature, non-infectious virions are assembled in the ER, where viral RNA is complexed with protein C and packaged into an ER-derived lipid bilayer containing heterodimers of prM and E proteins (Mackenzie and Westaway, 2001; Lorenz et al., 2003). The prM protein acts as a scaffold to prevent the virus from fusing prematurely during transport out of the cell (Li et al., 2008). Subsequently, PrM is cleaved into M by the cellular protease furin in the trans-Golgi network (Yu et al., 2008). Finally, mature infectious virus particles are released outside the cell by exocytosis (Figure 2).

## Apoptosis

Apoptosis, also known as programmed cell death (PCD), is a self-protection mechanism used by multicellular organisms to eliminate senescent, damaged, or pathogen-infected cells. Apoptosis is regulated by two classical signaling pathways: the intrinsic and the extrinsic apoptotic pathways. Apoptotic cells are manifested by cell shrinkage, chromatin aggregation, DNA fragmentation, mitochondrial swelling, and finally the formation of apoptotic bodies, which are cleared by phagocytes (Kennedy, 2015). Studies have found that apoptosis plays an important role in the pathogenesis of viral infections. As a key innate defense mechanism, it inhibits virus replication and clears virus-infected cells (Benedict et al., 2002), which is of great significance for maintaining the healthy development of organism and the normal function of the immune system (Liu et al., 1996). However, many viruses have evolved strategies to prevent or delay the occurrence of apoptosis during virus replication until sufficient progeny viruses are produced. Moreover, some viruses-induced apoptosis can enhance the spread of the virus, leading to tissue damage and disease. In this article, we focus on reviewing the roles played by flavivirus in the regulation of cell apoptosis, and understanding the mechanism of flavivirus regulating apoptosis will be helpful for future research.

### The Intrinsic Pathway

The occurrence of the intrinsic apoptosis signal pathway of apoptosis mainly involves various non-receptor-mediated stimuli, generating intracellular signals, and directly acting on intracellular target (mitochondria), leading to apoptosis. Stimulants that cause endogenous apoptosis act in an active or passive manner. Passive signals include the loss of specific growth factors, hormones and cytokines, leading to an unlimited death program, thereby triggering cell apoptosis (Papaianni et al., 2015). Other stimuli that act in an active manner include but are not limited to radiation, toxins, hypoxia, high fever, viral infection, and free radicals.

The mitochondrial pathway in apoptosis is mainly regulated by members of the Bcl-2 family proteins (Ashkenazi and Salvesen, 2014; Papaianni et al., 2015). Bcl-2 family proteins have pro-apoptotic or anti-apoptotic effects. So far, 25 genes have been identified in the Bcl-2 family. Anti-apoptotic proteins include Bcl-2, Bcl-X_*L*_, Bcl-x, Bcl-XS, Bcl-w, and BAG, pro-apoptotic proteins include Bax, Bak, Bid, Bad, Bim, Bcl-10, Bik, and Blk (Wong and Puthalakath, 2008; Kilbride and Prehn, 2013; Borner and Andrews, 2014). These proteins all play a role in determining whether a cell undergo apoptosis. Studies have found that the main mechanism of Bcl-2 family protein function is to modulate the mitochondrial membrane permeability to regulate the release of cytochrome c (Cyt-c).

**FIGURE 3:**
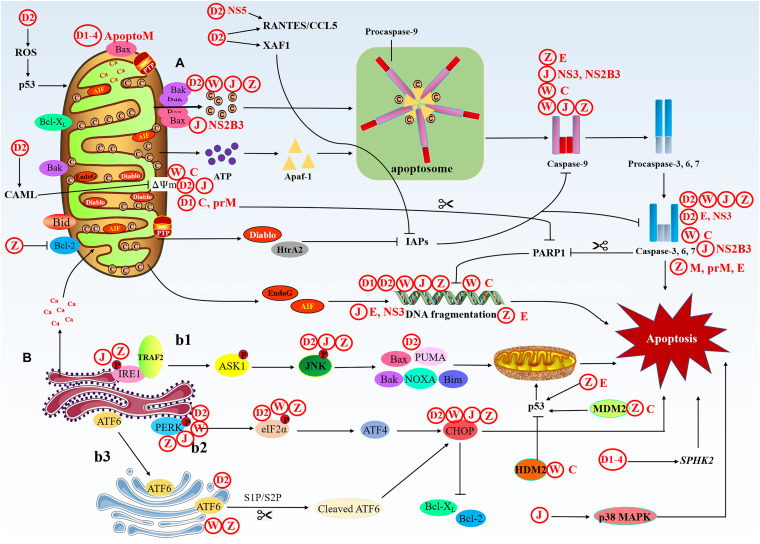
The intrinsic apoptotic pathway. The intrinsic cell death pathway is activated by an intracellular death signal. **(A)** This signal results in the oligomerization and translocation of Bak and Bax into the outer membrane of the mitochondria. This triggers mitochondrial outer membrane permeabilization (MOMP) and the release of Cyt-c and IAP binding proteins. Cyt-c forms a complex with pro-caspase-9 and Apaf-1, leading to activation of caspase-9, then caspase-9 activates the executioner caspases (caspase-3, -6, and -7) and induces cell death. The IAP binding proteins, such as Diablo and HtrA2, enhance caspase activation through the neutralization of IAPs. **(B)** Under ER stress, three upstream signaling proteins, IRE1, PERK, and ATF6, are activated, thus leading to a cascade of activity that induces apoptosis. (b1) The prolonged activation of IRE1 can promote apoptosis. Phosphorylated IRE1 recruits TRAF2 and triggers a cascade of phosphorylation events, such as the activation of ASK1, which ultimately phosphorylates and activates JNK. Then, JNK phosphorylation activates pro-apoptotic genes and induces apoptosis through the mitochondrial pathway. (b2) The homomultimerization and autophosphorylation of PERK leads to eIF-2α phosphorylation, which increases the translation of ATF4. Then, ATF4 upregulates the expression of CHOP, which promotes apoptosis. (b3) ATF6 migrates to the Golgi apparatus to undergo cleavage, first by S1P and then by S2P, cleaved ATF6 can also promote apoptosis via upregulation of CHOP.

The stimulus causes changes in the inner mitochondrial membrane, leading to the opening of the permeability transition pore (PTP), the loss of mitochondrial membrane potential (MMP), and the release of two groups of pro-apoptotic proteins from the membrane space into the cytoplasm (Tait and Green, 2010; Figure 3). The first group contains Cyt-c, DIABLO/Smac and serine protease HtraA2/Omi (van Loo et al., 2002; Garrido et al., 2006). These proteins activate the caspases-dependent mitochondrial pathway, cyt-c binds and activates apaf-1 and procaspase-9 to form “apoptosome” (Yuan and Akey, 2013). The accumulation of procaspase-9 leads to the activation of caspase-9, and caspase-9 triggers the caspase cascade, then the activated caspase-3/6/7 executes apoptosis. DIABLO/Smac and serine protease HtrA2/Omi have been reported to inhibit IAPs (inhibitors of apoptotic protein) and promote apoptosis (Schimmer, 2004). The second group of pro-apoptotic proteins includes AIF (apoptosis-inducing factor) and EndoG (endonuclease G), and the functions of AIF and EndoG are independent of caspases. These two proteins are released from the mitochondria when apoptosis occurs, and as a late event, it occurs in the dying cells. AIF translocate into the nucleus, causing DNA fragmentation and condensation of peripheral nuclear chromatin (Joza et al., 2001). EndoG can also be translocated into the nucleus to cut-off nuclear chromatin and produce oligomeric nucleosome DNA fragments (Jang et al., 2015). As a site for protein synthesis and processing, the ER is imbalanced due to physical and chemical stimulation or infection of pathogenic microorganisms, resulting in the inability of protein folding or misfolding. When these conformationally dissimilated proteins accumulate in the ER and cannot be effectively eliminated, the cells will initiate an unfolded protein response (UPR). Long-term activation of UPR can trigger cell apoptosis through IRE1 (inositol requiring protein-1), PERK (protein kinase RNA-like ER kinase), and ATF6 (activating transcription factor-6) pathways, which are connected with mitochondria-mediated apoptosis pathway (Verma and Datta, 2012). First, phosphorylated IRE1 recruits TRAF2 (TNF receptor-associated factor 2) and triggers a cascade of phosphorylation events, such as the activation of ASK1 (apoptosis signaling kinase 1), and ultimately phosphorylates and activates JNK. Then, JNK activates pro-apoptotic genes Bax, Bak, Bim, PUMA, and NOXA, these genes are transferred to the mitochondrial to initiate apoptosis (Upton et al., 2012). The homomultimerization and autophosphorylation of PERK lead to phosphorylation of eIF2α (eukaryotic translation initiation factor 2α), which increases the translation of ATF4 (activating transcription factor 4). Then, ATF4 upregulates the expression of CHOP (C/EBP-homologous protein) and activates apoptosis (Tabas and Ron, 2011). CHOP can reduce the expression of anti-apoptotic genes Bcl-2 and Bcl-X_*L*_ (Puthalakath et al., 2007). ATF6 binds to the ER membrane, but when protein homeostasis is disrupted, ATF6 then migrates to the Golgi apparatus to undergo cleavage, first by Site 1 Protease (S1P) and then by Site 2 Protease (S2P) (Ye et al., 2000), cleaved ATF6 can also promote cell apoptosis via upregulating of CHOP (Morishima et al., 2011; Hirsch et al., 2014).

### The Extrinsic Pathway

**FIGURE 4:**
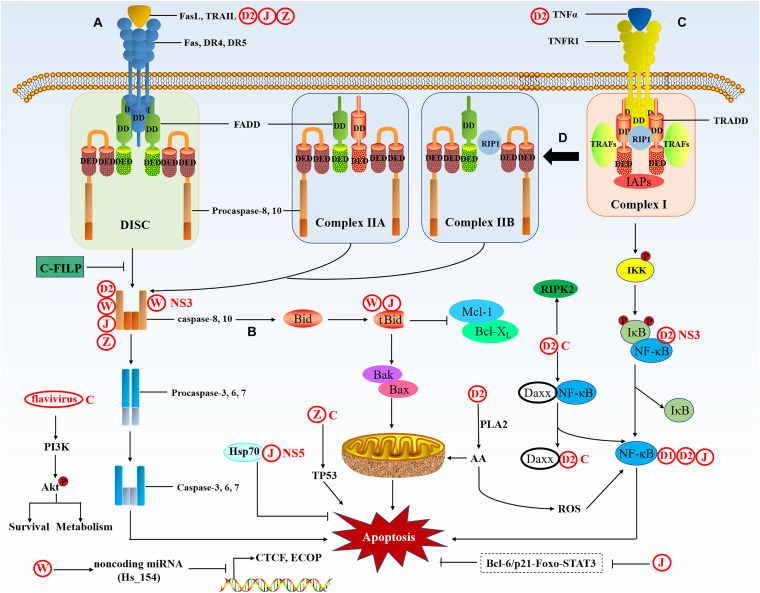
The extrinsic apoptotic pathway. The extrinsic pathway is activated by death signals mediated by death ligands. **(A)** Fas and DR4/5 are activated by the binding of their respective ligands FasL and TRAIL; the receptors then bind to FADD via the DD. Then, the DED of FADD binds to procaspase-8/10, which forms a complex called the DISC and sends a signal to activate caspase-8 and -10, then caspase-8 and -10 induce the activation of the caspase cascade and ultimately results in apoptosis. **(B)** In a particular cell, the extrinsic pathway can crosstalk with the intrinsic pathway through caspase-8-mediated truncation of Bid to tBid. tBid activates BAK/BAX oligomerization and induces apoptosis through the mitochondrial pathway. **(C)** TNFR1 is activated by the binding of its respective ligand TNFα. TNFR1 recruits TRADD, an adaptor protein that binds to TRAFs, RIP1, and IAPs, forming the initial membrane complex (complex I), which stimulates the NF-κB pathways to facilitate apoptosis. **(D)** Complex I forms two types of cytoplasmic apoptotic complexes, TRADD-dependent complex IIA and RIP1-dependent complex IIB, which activate caspase-8, thus initiating apoptosis.

The extrinsic apoptotic pathway is mediated by specific death receptors (DRs) on the cell membrane, the DRs are members of the TNF (tumor necrosis factor) superfamily (Locksley et al., 2001). The death domain (DD) of DRs transmits death signals from the cell surface to the intracellular signaling pathway. So far, ligands and corresponding DRs include FasL/Fas, TNF-α/TNFR1, Apo3L/DR3, Apo2L/DR4, and Apo2L/DR5. When the ligand binds to the receptor, it recruits cytoplasmic linker protein and binds to the receptor through DD (Suliman et al., 2001; Rubio-Moscardo et al., 2005). Taking the FasL/Fas model as an example, the binding of FasL (Fas ligand) to Fas can recruit the linker protein FADD (Fas-associated protein with death domain) through the DD (Wajant, 2002; Figure 4). Then FADD combined with procaspase-8/10 through the dimerization of the death effector domain (DED) to form a death-inducing signaling complex (DISC), leading to autoproteolytic cleavage of procaspase-8/10 (Kischkel et al., 1995). The activated caspase-8/10 have enzymatic activity and can hydrolyze caspase-3/6/7 to induce apoptosis (Ashkenazi, 2008). There is a related cross-connection between the DR (extrinsic) pathway and the mitochondrial (intrinsic) pathway. In certain cells, activated caspase-8 cleaves the pro-apoptotic protein Bid to create truncated Bid (tBid), thereby activating the mitochondria-mediated apoptosis signaling pathway (Huang K. et al., 2016). TNFR1 is activated by the binding of its respective ligand TNF. TNFR1 recruits TRADD, an adaptor protein that can bind to TNF receptor-associated factors (TRAFs), receptor-interacting protein kinase (RIP1) and cellular IAPs to form an initial membrane complex (complex I), which stimulates the NF-κB pathway to facilitate cell apoptosis (Mahmood and Shukla, 2010). The initially formed TRADD-RIP1-TRAFs complex I then assembles into the cytoplasmic apoptotic complex (complex II). There are two types of cytoplasmic complex II, TRADD recruits FADD and caspase-8 to form TRADD-dependent complex IIA, RIP1 recruits FADD and caspase-8 to form complex IIB, and cIAPs can negatively regulate complex IIB. Both complex IIA and IIB can trigger apoptosis by activating caspase-8 (Galluzzi et al., 2012; Pobezinskaya and Liu, 2012).

## Apoptosis During Flavivirus-Infection

### Dengue Virus

#### DENV Regulation of Apoptosis

Dengue virus is a serious arbovirus and the most lethal among all Flavivirus members. There are four distinct DENV strains (DENV 1–4) in serology. Dengue infection may cause diverse pathogenic conditions, ranging from mild-flu like febrile syndrome (dengue fever) to very serious conditions caused by infection with the second serotype, the dengue hemorrhagic fever (DHF) or the dengue shock syndrome (DSS) (Courageot et al., 2003).

##### DENV1

Previous studies have shown that DENV1 infection can lead to typical apoptosis in a variety of human and animal cells including BHK, HUH-7, Vero, and mouse neuroblastoma (Neuro 2a) cell lines (Desprès et al., 1996; Nasirudeen et al., 2008). And DENV1 replicated in human hepatoma cells (HepG2) leads to the activation of transcription factor NF-κB and induces apoptosis (Marianneau et al., 1997). Another study found that accumulation of DENV1 viral protein in the ER, rather than the release of virus, can induce ER stress and activate the apoptosis pathway. In addition, DENV1 C and prM proteins have been shown to trigger MMP down-regulation and p53 expression in HUH-7 cells, suggesting that mitochondria- and p53-mediated pathways are very likely to participate in DENV1-induced apoptosis (Nasirudeen et al., 2008). The regulatory effects of DENV and its proteins on apoptosis are summarized in Table 1.

**TABLE 1:** Pro-apoptotic or anti-apoptotic activity of DENV and its proteins.

**Virus**	**Viral protein**	**Strains**	**Tested cell lines**	**Function**	**References**
DENV1		Oster	HepG2	Pro-apoptotic	Marianneau et al., 1997
		FGA/89, BR/90	Neuro 2a		Desprès et al., 1996
		Hawaii	HUH-7		Morchang et al., 2017
			BHK, HUH-7, Vero		Nasirudeen et al., 2008
	C, prM		HUH-7		
	M	FGA/89	Neuro 2a, HepG2		Catteau et al., 2003
DENV2			HMEC-1, ECs	Pro-apoptotic	Limonta et al., 2007; Ochoa et al., 2009; Lin et al., 2014
		Asiatic 16681	HepG2, ECV304, monocytes, HUH-7		Avirutnan et al., 1998; El-Bacha et al., 2007; Torrentes-Carvalho et al., 2009; Thepparit et al., 2013; Morchang et al., 2017; Sreekanth et al., 2017
		New Guinea C	HUVECs, EA.hy926		Liao et al., 2010; Long et al., 2013; Huang et al., 2014
		New Guinea C, Asiatic 16681	Mo-DC		Olagnier et al., 2014
		PL046	SK-N-SH		Jan et al., 2000
		New Guinea C	HUH-7, C6/36	Anti-apoptotic	Yu et al., 2006; Li et al., 2012
		PL046	2fTGH, N18		Yu et al., 2006; Limjindaporn et al., 2007; José and Eva, 2011; Morchang et al., 2011
	C	Asiatic 16681	HepG2	Pro-apoptotic	Netsawang et al., 2010; Amar et al., 2011
		New Guinea C	DIV2 Neurons, SH-SY6Y		Slomnicki et al., 2017
	M	Jamaica	Neuro 2a, HepG2		Catteau et al., 2003
	EIII	PL046	MKs		Lin G.-L. et al., 2017
	NS3, NS2B-NS3		HMEC-1		Ochoa et al., 2009; Lin et al., 2014
		New Guinea C	Vero		Shafee and AbuBakar, 2003
DENV3		DENV3/5532, DENV3/290	mdDCs	Pro-apoptotic	Silveira et al., 2011
		H87	HUH-7		Morchang et al., 2017
	M		Neuro 2a, HepG2		Catteau et al., 2003
DENV4		H241	HUH-7	Pro-apoptotic	Morchang et al., 2017
	M		Neuro 2a, HepG2		Catteau et al., 2003

##### DENV2

###### Pro-apoptosis

Autopsy of fatal DHF/DSS cases caused by DENV2 demonstrated apoptotic cells in liver, brain, intestinal and lung tissues, and the apoptotic microvascular endothelial cells (ECs) in intestinal and pulmonary tissues explain the patient’s plasma leakage (Limonta et al., 2007). DENV2 infection of human ECs leads to up-regulation of IL-8 (interleukin-8) and RANTES (regulated upon activation normal T cell expressed and secreted factor), these cytokines accumulate at serosal sites and cause local vascular leakage, which has been observed to accompany apoptosis (Avirutnan et al., 1998). Further research found that DENV2 induces ECs apoptosis through XIAP-associated factor 1 (XAF1)-dependent pathway, XAF1 is one of the interferon-inducing genes, it upregulates caspase-3 36 h after infection and mediates cell apoptosis (Long et al., 2013; Huang et al., 2014).

Besides, the intrinsic apoptotic pathway also plays an important role in this context. In HepG2 cells, DENV2 promoted changes in mitochondrial bioenergetics and caused typical apoptotic morphological changes, including cytoplasmic contraction, mitochondrial swelling, and plasma membrane blistering (El-Bacha et al., 2007). In fact, changes in mitochondrial promote the activation of caspase-9, which then activates caspase-3, leading to DNA cleavage and apoptosis (Torrentes-Carvalho et al., 2009; Suwanmanee and Luplertlop, 2017). Further results showed that in HepG2 cells, DENV2 initiated the UPR and the Noxa/PUMA stress response pathways (Thepparit et al., 2013). Moreover, a series of protein phosphorylation reactions are essential for inducing cell apoptosis. For example, DENV2 (strain 16881) increases the phosphorylation of JNK1/2 and p38MAPK through both intrinsic and extrinsic apoptotic pathways, leading to liver injury. The induced p53 phosphorylation and the decrease of anti-apoptotic Bcl-2 expression indicate the involvement of intrinsic apoptotic pathway, in which the induced expression of TNF-α and TRAIL may participate in the extrinsic apoptotic pathway (Sreekanth et al., 2017).

In addition, DENV2 can activate the transcription factor NF-κB, NF-κB translocates to the nucleus to activate pro-inflammatory cytokine TNF-α, and TNF-α binds to its transmembrane receptor TNFR to activate caspase-8 and downstrem effector caspase-3 (Amar et al., 2011; Netsawang et al., 2014). Similarly, FasL interacts with Fas receptor present on immune cells to trigger apoptosis signals (Liao et al., 2010). In the TNF-α and FasL signaling pathways, the activation of enzyme phospholipase A2 (PLA2) seems to be essential for inducing apoptosis (Jäättelä et al., 1995; Nevalainen and Losacker, 1997). This enzyme converts membrane phospholipids into arachidonic acid (AA), which is the main lipid mediator of several intracellular reactions (Malewicz et al., 1981; Axelrod et al., 1988). AA stimulates the synthesis of NADPH oxidase to produce superoxide anions and other reactive oxygen species (ROS) (Jan et al., 2000), ROS can act as signal transducers and activate the expression of genes involved in apoptosis like NF-κB (Xiao and Ghosh, 2005). As well, excessive ROS will eventually initiate the mitochondrial apoptosis pathway (Olagnier et al., 2014). Moreover, a positive correlation between apoptosis, DNA damage and oxidative stress was found in PBMCs infected with DENV2, which paved the way for the determination of plausible immunopathological links contributing to disease pathogenesis. Besides, secondary messenger oxides like nitric oxide (NO) also mediates dengue-triggered apoptosis in a caspase dependent manner (Lin C.-F. et al., 2002). Beyond that, functional studies have shown that knocking down the sphingosine kinase2 (SPHK2) in HUH-7 cells infected with four serotypes of DENV reduce the activities of caspase-9 and caspase-3, suggesting that SPHK2 plays a role in promoting apoptosis through intrinsic pathway (Morchang et al., 2017).

###### Anti-apoptosis

Although DENV2 has the function of inducing cell apoptosis, it also has the function of inhibiting cell apoptosis. José and Eva (2011) demonstrated that DENV2 manipulates the sequence of events to activate and inhibit the three different branches of UPR in a time-dependent manner, so that cells can adapt to infection stress, overcome translational inhibition, prevent premature apoptosis, and ultimately extend the life cycle of the virus. For instance, inhibition of XBP1 combined with DENV2 infection can lead to weakened ER expansion, enhanced cytopathic effects of the virus and increased levels of the apoptosis marker procaspase-3 (Yu et al., 2006). This further proves that the IRE1-XBP1 pathway can protect cells from apoptosis and reduce ER stress contributing to DENV pathogenesis, similar results have been observed in JEV-infected cells. Moreover, in mosquito cells, DENV-2 infection causes UPR to activate the PERK signaling pathway, thereby reducing the accumulation of ER stress, and activating anti-apoptotic effects to help cells survive the continuous amplification of the virus (Hou et al., 2017). This phenomenon is very important for elucidating how mosquitoes can healthily serve as carriers of DENV and may-be other arboviruses. Another result showed that all the capsid proteins from six different flaviviruses (DENV, JEV, WNV, YFV, MVEV, and SLEV) confer a protective effect on Fas-dependent apoptosis in a manner that increases phosphorylation of Akt thereby enhancing cell activity (Hart and Vogt, 2011). Moreover, protein phosphatase 1, which is known to inactivate Akt, was identified as a DENV-C interacting protein, indicating that DENV-C activates Akt by sequestering phosphatases that downregulate phosphor-Akt (Airo et al., 2018).

Meanwhile, some factors play a role in inhibiting apoptosis in the process of DENV infection. DENV2-infected cells express high levels of calcium modulating cyclophilin-binding ligand (CMAL), a regulator of intracellular calcium levels (Bram and Crabtree, 1994), the cells therefore have high cytosolic calcium concentration, which can help DENV2 to subvert apoptosis since it protects cells from mitochondrial damage (Li et al., 2012). Bcl-xL also plays a vital role in the survival of DENV, JEV, and ZIKV infected cells, therefore Bcl-xL provides a novel antiviral target for inhibiting the propagation of flavivirus (Suzuki et al., 2018).

#### Regulation of Apoptosis by DENV Proteins

Limjindaporn et al. (2007) confirmed that the DENV2 capsid protein (DENV2-C) physically interacts with death protein 6 (Daxx), a human DD-associated protein, and the nuclear localization of DENV2-C is closely related to DAXX interaction and apoptosis induction, elucidating the pro-apoptotic function of DENV2-C (Netsawang et al., 2010). Moreover, DENV2-C promotes the release of NF-κB by binding Daxx, which usually interacts with NF-κB. Since NF-κB can activate CD137 promoter, the interaction between CD137 ligand and CD137 eventually activates caspase cascades and apoptosis, indicating that NF-κB signal contributes to DENV2-C-induced apoptosis (Nagila et al., 2011). Cell death gene RIPK2 (receptor-interacting serine/threonine protein kinase 2), is a key mediator of various stress responses, leading to the activation of NF-κB, MAPK, and caspases. The expression of RIPK2 contributes to apoptosis induced by capsid protein but only for DENV-2 and -4 serotypes (Morchang et al., 2011). There is evidence that the M proteins of four DENV serotypes can trigger the intrinsic apoptosis pathway in host cells such as Neuro 2a and HepG2 cells, and the nine carboxy-terminal amino acids (residues M-32 to M-40) of M protein called ApoptoM is cytotoxic and directly involved. In addition, the M ectodomains of JEV, WNV, and YFV also have pro-apoptotic effects, indicating that the M protein plays a key role in determining the fate of flavivirus-infected cells (Catteau et al., 2003). Another study found that DENV2-envelope protein domain III (DENV2-EIII) suppresses megakaryopoiesis by inducing apoptosis of differentiated Megakaryocytes (MKs), which is one of the causes of thrombocytopenia (Lin G.-L. et al., 2017). Therefore, DENV2-EIII can be used as a drug target to for the treatment of MKs deficiency and thrombocytopenia in severe dengue.

Subsequently, Ochoa et al. (2009) found that human microvascular endothelial cells (HMEC-1) transfected with DENV2 serine protease NS3 or NS2B-NS3 complex can trigger apoptosis. Further study have shown that NS3 activates NF-κB by inducing IκBα/IκBβ cleavage and activating IκB kinase, which triggers the extrinsic apoptotic pathway (Lin et al., 2014). Moreover, NS2B-NS3 caused a higher proportion of apoptosis, indicating that NS2B is an important cofactor for NS3 to induce apoptosis (Shafee and AbuBakar, 2003). Interestingly, DENV-2 NS5 can also translocate into nucleus and interact with Daxx to further increase the production of RANTES. Therefore, NS5 may have a similar apoptosis-inducing function to DENV2-C, but this requires further experimental verification (Nagila et al., 2011; Khunchai et al., 2012).

### West Nile Virus

#### WNV Regulation of Apoptosis

Although WNV is not as versatile as dengue, it can trigger central nervous system (CNS) cell apoptosis, therefore establishing neuroinvasiveness. Neuro-2a cells (neuronal cells) and K562 (immune cells) infected by WNV show typical apoptotic characteristics, and the up-regulated Bax gene is involved (Parquet et al., 2001). Furthermore, WNV infection can induce T98G cells (brain-derived) death through extrinsic and intrinsic apoptotic pathways, and caspase-3 dependent apoptosis contributes to the pathogenesis of fetal WNV encephalitis (Bimmi et al., 2003; Kleinschmidt et al., 2007; Samuel et al., 2007; Peng and Wang, 2019), which helps to clarify the pathogenesis of WNV-induced neuronal cell death and propose novel therapeutic targets that may restrict CNS damage. In Vero cells (epithelial cells), WNV can trigger the mitochondrial-mediated apoptosis pathway at low infectious doses (MOI < 10), which is initiated by the release of Cyt-c and the formation of apoptosome. In contrast, cells infected with a higher load (MOI > 10) showed signs of necrosis (Chu and Ng, 2003). The regulation of WNV and its protein on apoptosis is summarized in Table 2.

**TABLE 2:** Pro-apoptotic or anti-apoptotic activity of WNV and its proteins.

**Virus**	**Viral protein**	**Strains**	**Tested cell lines**	**Function**	**References**
WNV		Eg101	K562, Neuro-2a	Pro-apoptotic	Parquet et al., 2001
		3000.0259 strain	ESNC		Bimmi et al., 2003; Samuel et al., 2007
		NY385-99 strain	T98G, SK-N-MC, HEK293T, MEFs		Kleinschmidt et al., 2007; Medigeshi et al., 2007
		Sarafend strain	Vero		Chu and Ng, 2003
		9935262 strain	Midgut epithelial cells of mosquitoes		Vaidyanathan and Scott, 2006
		NY-99 strain	HeLa, 293, RD, SH-SY5Y		Joo-Sung et al., 2002
	C		MEFs, SH-SY5Y	Pro-apoptotic	Yang et al., 2010
		Sarafend strain	BHK		Bhuvanakantham et al., 2010
		NY835-99 strain	A549, HEL/18	Anti-apoptotic	Urbanowski and Hobman, 2013
		NY-99 strain	A549		Airo et al., 2018
	M	IS-98-ST1	Neuro 2a, HepG2	Pro-apoptotic	Catteau et al., 2003
	NS2A	Kunjin strain	Vero76 cells		Melian et al., 2013
	NS3	Merion strain	RD, Neuro-2a, Vero, Hela, NIH3T3		Ramanathan et al., 2006
	NS2B-NS3		RD, Neuro-2a, Vero		

The ER stress is also involved, Medigeshi et al. (2007) found that the activation of two branches of UPR (ATF6 and PERK) after WNV infection can induce the production of proapoptotic CHOP and downstream apoptosis. However, WNV_*KUN*_ (Kunjun strain) closes PERK pathway while activates the remaining two UPR (ATF6 and IRE1) pathways in MEFs, further studies have found that these pathways increase cell viability and viral load by restricting apoptotic cell death (Ambrose and Mackenzie, 2013). Surprisingly, after the WNV vector mosquitoes *Culex pipiens pipiens* (*C.p.pipiens*) are infected with WNV, a large number of midgut epithelial cells undergo apoptosis, which may affect virus transmission, disease epidemiology and viral evolution (Vaidyanathan and Scott, 2006). Furthermore, in human and mouse cell culture, WNV affects non-coding microRNAs (miRNAs), among which microRNA Hs_154 is significantly upregulated. Two of its targets, CTCF (CCCTC-binding factor) and ECOP (EGFR-coamplified and overexpressed protein) are related to cell survival (Smith et al., 2012). The observation suggests that induction of Hs_154 expression can promote cell apoptosis, so this miRNA may become a candidate for therapeutic intervention.

#### Regulation of Apoptosis by WNV Proteins

Joo-Sung et al. (2002) demonstrated for the first time that transfection of WNV-C in neuronal and non-neuronal cells can cause inflammation and apoptosis. Further studies have shown that WNV-C induces apoptosis by destroying mitochondrial membranes and activating caspase-9 and caspase-3, and the 3′-terminal regions of the capsid protein is related to its apoptosis-inducing function. In addition, the WNV-C protein phosphorylated by protein kinase C (PKC) can enhance its binding and nucleus co-localization with HDM2 protein, a p53 negative regulator, thereby stabilizing p53 and then inducing its apoptosis target protein Bax (Bhuvanakantham et al., 2010; Yang et al., 2010). Despite the substantial evidence supporting the pro-apoptotic effect of capsid protein, Urbanowski and Hobman (2013) found that WNV-C inhibits cell apoptosis by activating Phosphatidylinositol-3-kinase (PI3K)-Akt pro-survival pathway in four mammalian cells (A549, HEK293T, Vero-76, and BHK-21). The discrepancy between these reports may be due to the capsid protein containing an 18-amino-acid-residue signal peptide of prM in the earlier reports.

Liu et al. (2006) mutated the alanine at position 30 of the WNV-NS2A protein to proline (A30P) and found that the virulence of the virus was significantly weakened. Further, Melian et al. (2013) used this mutant and wild strain to infect IFN-β-deficient cells, and found that the DNA degradation of the mutant group was reduced and the number of TUNEL-labeled positive cells decreased significantly, indicating that the WNV-NS2A protein plays a role in IFN-independent apoptosis. Moreover, the expression of NS2B-NS3 or NS3 protein, but not NS2B, can induce apoptosis. Further studies have showed that WNV-NS3 can trigger apoptosis mediated either individually or together by the caspases-8 and -3 pathways, and the protease and helicase domains of NS3 protein are both essential for inducing apoptosis (Ramanathan et al., 2006). The thorough knowledge of the apoptotic pathways driven by NS2B-NS3 and NS3 will undoubtedly provide valuable insights for development of novel therapeutics for this viral infection.

### Japanese Encephalitis Virus

#### JEV Regulation of Apoptosis

Although the pathogenic mechanism of JEV is similar to that of WNV, JEV manipulates both intrinsic and extrinsic pathways to its advantage. The replication of JEV triggers a variety of cell apoptosis, as confirmed by DNA fragmentation ladder, nuclear condensation and TUNEL assay (Liao et al., 1997), and the stable expression of human Bcl-2 can delay JEV-induced apoptosis (Liao et al., 1998). And the presence of apoptosis was validated in the subventricular zone of JEV infected BALB/c mice, which was companied by the significant increase of caspase proteins (Mukherjee et al., 2017). Although JEV induces the classic intrinsic apoptotic pathway in N18 neuroblastoma cells, it activates both caspase-8 (part of the extrinsic pathway) and caspase-9 in a predominantly mitochondria-dependent pathway in MCF cells (Tsao et al., 2008). Interestingly, in addition to apoptosis, JEV can also activate other PCD pathways, especially the pyroptosis and necroptosis pathways, which were verified by the immunofluorescent staining of specific markers (Wang et al., 2020). The regulation of JEV and its protein on apoptosis is summarized in Table 3.

**TABLE 3:** Pro-apoptotic or anti-apoptotic activity of JEV and its proteins.

**Virus**	**Viral protein**	**Strains**	**Tested cell lines**	**Function**	**References**
JEV			hNS1	Pro-apoptotic	Mukherjee et al., 2017
		RP-9	BHK-21, N18, NT2, MCF7, CHO cells		Hong-Lin et al., 2002; Tsao et al., 2008
		SA14-14-2	BHK-21		Huang M. et al., 2016
		P3	Neuro-2a		Lin et al., 2004
		T1P1	TE671		Yang et al., 2009
		JEV-YL	HepG2, Vero		Chen et al., 2006
		RP-9	N18	Anti-apoptotic	Yu et al., 2006
	C		A549	Anti-apoptotic	Airo et al., 2018
	M	Nakayama	Neuro 2a, HepG2	Pro-apoptotic	Catteau et al., 2003
	E	JEV-YL	HepG2, Vero		Chen et al., 2006
	NS3		Vero, HeLa		Yiang et al., 2013
	NS2B-NS3	T1P1	TE671		Yang et al., 2009
	NS5			Anti-apoptotic	Weng et al., 2018

Further, the apoptotic cell death induced by JEV depends on ER stress and the generation of ROS. Current research shows that these three proteins GRP78, mitochondrial protein Prohibitin (PHB), and heterogeneous nuclear ribonucleoprotein (hnRNPC) interact with JEV RNA, which in turn causes ER stress-induced apoptosis (Mukherjee et al., 2017). Another result shows that JEV-induced ER stress participates in the apoptosis process through p38-dependent and CHOP-mediated pathways (Hong-Lin et al., 2002; Sankar et al., 2014), and the IRE1/JNK pathway of ER stress is also an important mechanism for JEV to induce apoptosis (Huang M. et al., 2016). Even replication-incompetent strain (UV-JEV) retain their ability to kill neuronal cells by triggering a ROS-dependent, partly NF-κB-mediated pathway (Lin et al., 2004).

In addition, Guo et al. (2018) found that JEV induces apoptosis by inhibiting the STAT3-Foxo-Bcl-6/p21 pathway, indicating that this pathway has a certain pro-survival effect, which provides novel insights into JEV-induced encephalitis. Followed, significant up-regulation of Bax, Bid, Fas, and FasL and down-regulation of IGFBP-2, p27, and p53 were respectively observed in JEV infected cells with 0.5 and 10 MOI compared to uninfected cells (Al-Obaidi et al., 2017). As a result, in the case of low MOI, expression of proteins involved in inducing apoptosis indicates that the immune system has played a protective role of in preventing the virus from completing its replication and producing infectious progeny viruses. While at high MOI, most of proapoptotic proteins are reduced, which means that the virus has capacity to disable host cell apoptotic mechanisms that may be obligatory for virus life cycle completion.

#### Regulation of Apoptosis by JEV Proteins

Firstly, E protein of JEV can induce apoptosis of HepG2 and Vero cells (Chen et al., 2006). Further studies have shown that JEV NS3 protein, as well as NS3 helicase and protease domains induce apoptosis through caspase-dependent (caspase-9/-3) and -independent pathways (Yiang et al., 2013). Research by Yang et al. (2009) showed that JEV NS2B-NS3 induces the reduction of MMP and the release of Cyt-c, leading to mitochondrial-mediated apoptosis. Moreover, ROS-mediated activation of ASK1-p38 MAPK signaling pathway is also related to NS2B-NS3 induced apoptosis. Recently results show that JEV-NS5 not only participates in type I IFN antagonism, but also plays an anti-apoptotic effect by preventing IFN-β-induced p38 MAPK/STAT1-mediated apoptosis (Weng et al., 2018).

### Zika Virus

#### ZIKV Regulation of Apoptosis

It is worth noting that ZIKV infection can induce apoptosis through caspase-3-mediated pathways both *in vitro* and *in vivo* (Huang W.-C. et al., 2016; Chen et al., 2017; Lin M.-Y. et al., 2017; Yan et al., 2019). For example, ZIKV infection in mouse kidneys caused caspase-3-mediated apoptosis of renal cells (Chen et al., 2017). Newly research showed that infection of female rhesus monkeys early in pregnancy with ZIKV exhibited apoptosis of neuroprogenitor cells (Martinot et al., 2018). Further, a variety of apoptosis markers were detected in neural parenchyma isolated from clinical cases, including FASL, FAS, Bax, and caspase-3 (de Sousa et al., 2018). And numerous reports show that neural cell apoptosis increases after ZIKV infection (Huang W.-C. et al., 2016; Li et al., 2016; Qian et al., 2016; Souza et al., 2016; Ghouzzi et al., 2017). For example, ZIKV preferentially induces apoptosis of neuro progenitor cells (NPCs), which is confirmed by the activation of caspases-3/7, -8, and -9 and the significantly elevated gene expression of TRAIL, as well as ultrastructural and flow cytometry analysis (Tang et al., 2016; Jungmann et al., 2017; Lee et al., 2020). In human neural stem cells (hNSCs), the cleavage of PARP and caspase-3 are participated in the apoptosis process (Devhare et al., 2017). New study observed mitochondrial fragmentation and disrupted MMP after 24 h of ZIKV infection in human neural stem cells (NSCs) and the SNB-19 glioblastoma cell line (Yang et al., 2020). These results contribute to ZIKV-induced abnormal development of the nervous system. The regulation of ZIKV and its protein on apoptosis is summarized in Table 4.

**TABLE 4:** Pro-apoptotic or anti-apoptotic activity of ZIKV and its proteins.

**Virus**	**Viral protein**	**Strains**	**Tested cell lines**	**Function**	**References**
ZIKV			Neural parenchyma	Pro-apoptotic	de Sousa et al., 2018
		KU940228	NPCs		Souza et al., 2016
		MR766	NPCs, RPTEpiCs, IPCs		Tang et al., 2016; Chen et al., 2017; Lin M.-Y. et al., 2017
		ZG-01	Renal cells		Liu T. et al., 2019
		ZIKV-LAV	GSCs		Chen et al., 2018
		Paraiba strain	Vero, A549		Park et al., 2019
		PF13, MR766, FSS13025	hNPCs		Zhang et al., 2016; Ghouzzi et al., 2017
		SZ01	CCF-STTG1, SK-N-SH		Tan et al., 2018
		ZIKA-BR	Placental tissues		Ribeiro et al., 2018
		PRVABC59	Huh7.5 and HepG2 cells		Sherman et al., 2019
		PRVABC59, MR766	hNSCs, hNPCs, NSCs, HTR-8, JEG-3 and JAR		Devhare et al., 2017; Lee et al., 2020; Yang et al., 2020; Muthuraj et al., 2021
		PRVABC59, Dakar, PF-25013-18	A549		Frumence et al., 2016; McFadden et al., 2018
		PRVABC59, FLR strain	HEK293		Liu H. et al., 2019
	C		U87 cells	Pro-apoptotic	Teng et al., 2017
		strain BeH819966	DIV2 Neurons, SH-SY5Y cells		Slomnicki et al., 2017
	M	strain BeH819015	A549, Huh7 cells		Vanwalscappel et al., 2020
	PrM	MR776	SNB-19 cells		Li et al., 2019
	E		fNSCs		Bhagat et al., 2018
		Haiti strain	PC12 cells		Liu et al., 2018
	NS4B		SH-SY5Y cells		Han et al., 2021
					

In addition to neural cells, many other cell types can also cause extensive apoptosis after ZIKV infection, such as HEK293 (embryonic cells) (Liu H. et al., 2019), GSCs (glioma stem cells) (Chen et al., 2018), Vero and A549 (Frumence et al., 2016; McFadden et al., 2018; Park et al., 2019) (epithelial cells) and hepatocyte cells lines (HuH7.5 and HepG2) (Sherman et al., 2019). Another result suggests that ZIKV induces renal apoptosis in both newborn and adult mouse models by down-regulating expression of Bcl-2 and the up-regulating the expression of cleaved caspase-3 and PARP (Liu T. et al., 2019), which is similar to that observed in neural cells. Interestingly, ZIKV infection is related to pro-inflammatory cytokine expression and apoptosis in placental explants, thus we propose that human placental explants can be used as a model for studying ZIKV infection *in vitro* (Ribeiro et al., 2018). Moreover, the mechanism of ZIKV-induced placental trophoblast apoptosis involves the activation of ER stress and JNK activation, and the inhibition of JNK dramatically prevents ZIKV-induced trophoblast apoptosis (Muthuraj et al., 2021).

The tumor suppressor protein p53 is also involved in ZIKV-mediated apoptosis, as the inhibition of p53 limits ZIKV-induced apoptosis in neural progenitors (Zhang et al., 2016). Since p53 can activate several pro-apoptotic genes such as Bax, Noxa and Puma, and inhibit anti-apoptotic gene survivin, leading to the activation of apoptosis. The physiological and metabolic changes in the ER after ZIKV infection lead to the activation of ER stress, such as initiating the IRE1-XBP1 and ATF6 pathways, and continuous ER stress then triggers apoptosis via upregulation of CHOP *in vivo* (Gladwyn-Ng et al., 2018; Tan et al., 2018; Oyarzún-Arrau et al., 2020).

#### Regulation of Apoptosis by ZIKV Proteins

Newly result indicate that ZIKV-C protein interacts with mouse double-minute-2 homolog (MDM2), which is involved in the p53-mediated apoptosis pathway, activating the death of infected neural cells, it also found that synthetic mimics of the ZIKV capsid protein induced cell death *in vitro* and *in vivo* (Teng et al., 2017). Further studies have shown that the nuclear localization of ZIKA-C is related to ribosomal stress (RS) and apoptosis, and the 22 C-terminal residues of ZIKV-C are essential for nuclear localization, RS and apoptosis (Slomnicki et al., 2017). Moreover, DENV2-C was found to have a similar apoptosis-inducing mechanism with ZIKA-C. New study demonstrates that the last C-terminal residues M-31/41 of the ZIKV M ectodomain have death-promoting activity through caspase-3/7 activation, which opens up attractive perspectives for ZAMP (Zika Apoptosis M Peptide) as an innovation anticancer agent (Vanwalscappel et al., 2020). ZIKV-prM protein can induce apoptotic cell death, and the Pr region located on the N-terminal side of prM protein is responsible for prM-induced apoptosis (Li et al., 2019). In addition, E protein of ZIKV can induce apoptosis in differentiating hNSC (Bhagat et al., 2018). ZIKV-E can also inhibit the proliferation of PC12 cells, trigger G2/M cell cycle arrest and apoptosis, further analysis showed that ZIKV-E caused apoptosis via intrinsic cell death pathway that was dependent on caspase-9/3 activation and accompanied by an increase in the ratio of Bax to Bcl-2 (Liu et al., 2018). The latest research shows that ZIKV and the NS4B protein of ZIKV can recruit Bax to the mitochondrial and induce cell apoptosis by activating the pro-apoptotic protein Bax, which is a new insight into understanding the interplay between apoptosis and ZIKV infection (Han et al., 2021).

### Other Flavivirus

Yellow fever virus infection increases the expression of caspase-3 and caspase-7, and ultimately leads to apoptosis (Holanda et al., 2019). Besides, YFV infection can regulate the expression of various cytokines such as TGF-β, TNF-α, IFN-β, and IFN-γ, which are participated in viral infection-induced apoptosis (Quaresma et al., 2006). Furthermore, the tick-borne flaviviruses LIV and TBEV can induce increased expression of apoptosis pathway related genes after infection *I*xodes *ricinus* cells (Mansfield et al., 2017). Langat virus can induce neural and non-neural cells apoptosis, further experiments showed that P35 protein can inhibit a variety of caspases, while LGTV E protein can activate caspase-3 by inhibiting the activity of P35 protein, thereby reducing cell viability and inducing apoptosis (Prikhod’Ko et al., 2001). The flavivirus duck tembusu virus (DTMUV) that infects poultry can also induce host cell duck embryo fibroblasts (DEFs) apoptosis and cell cycle arrest (Pan et al., 2020). The regulation of other flavivirus on apoptosis is summarized in Table 5.

**TABLE 5:** Pro-apoptotic or anti-apoptotic activity of other flavivirus and its proteins.

**Virus**	**Viral protein**	**Strains**	**Tested cell lines**	**Function**	**References**
YFV		BeH622205, BeH413820	HepG2 cell	Pro-apoptotic	Holanda et al., 2019
	C		A549 cells	Anti-apoptotic	Airo et al., 2018
	M	17D-204	Neuro 2a, HepG2	Pro-apoptotic	Catteau et al., 2003
LGTV		clone 636	Vero, LLC-MK2, Neuro-2a	Pro-apoptotic	Prikhod’Ko et al., 2001
	E		Vero, Neuro-2a		
TBEV		Neudorfl H2J	IRE/CTVM20	Pro-apoptotic	Mansfield et al., 2017
LIV		LI3/1		Pro-apoptotic	
MVEV	C		A549 cells	Anti-apoptotic	Airo et al., 2018
SLV					
DTMUV		CQW1 strain	DEFs	Pro-apoptotic	Pan et al., 2020

### Regulation of Apoptosis by Subgenomic Flaviviral RNA

Flaviviruses can utilize host mRNA degradation machinery to produce subgenomic flaviviral RNA (sfRNA). More and more studies have shown that sfRNA plays an important role in regulating cell apoptosis. For example, sfRNA is a determinant in DENV2-induced apoptosis and is possibly involved in PI3k/Akt signaling pathway through a Bcl-2-related mechanism, resulting in apoptotic cell death (Liu et al., 2014). Furthermore, sfRNA was shown to be required for WNV-induced neuropathogenicity in vertebrates as all mice infected with sfRNA-deficient virus failed to develop symptoms of encephalitis and survived the infection. Since both groups of animals had similar viral loads in the brain, the likely explanation for the lack of neuropathogenicity associated with the loss of sfRNA is the inability of the virus to induce apoptosis and kill infected brain cells (Pijlman et al., 2008). However, Chang et al. (2013) provided evidence that sfRNA suppresses the phosphorylation and translocated to the nucleus of IRF-3, which consequently inhibits IFN-β production and prevents apoptosis and, thus, contributes to viral persistence. Slonchak et al. (2020b) found that sfRNA inhibits apoptosis in the infected mosquitoes by altering expression of the genes that control cell death and survival, which was in line with the observed upregulation of caspase-7 during infection with sfRNA-deficient virus (Slonchak et al., 2020a). Moreover, the replication and transmission of sfRNA-deficient ZIKV mutants was restored by introduction of a caspase inhibitor. This demonstrates that the apoptosis pathway is a biologically relevant target of sfRNA and is responsible for viral attenuation associated with sfRNA deficiency. Understanding the role of sfRNA in flaviviruses infection may lead to future insights into the disease’s pathogenesis.

## Conclusion

This review is an in-depth understanding of flavivirus-induced apoptosis based on a wide array of reported work concerning flavivirus infection. It summarizes the current findings of flavivirus regulation of cell apoptosis, including a vast panel of distinct pro-apoptotic and anti-apoptotic mechanisms. The aftermath of virus invasion depends on the initial dose and cell type, and it can also switch to different mechanisms to exert its pathogenic effects in different cells. Taking the above viewpoints into consideration will help us better understand the mechanism of flavivirus-induced apoptosis and open a new gate for further research on the pathogenesis of flavivirus. And more emphasis needs to be put on studying the signaling pathways by which viruses regulate cell survival pathways. Future research should focus on the function of individual viral proteins, the host-virus interaction between viruses and cellular proteins, and the regulation of non-coding RNAs in viral infections.

## Author Contributions

YP contributed ideas for the review and wrote the manuscript and produced the figures. AC, MW, ZY, and RJ edited and revised the manuscript. All authors contributed to the article and approved the submitted version.

## Conflict of Interest

The authors declare that the research was conducted in the absence of any commercial or financial relationships that could be construed as a potential conflict of interest.

## Abbreviations

DENV: Dengue virus
JEV: Japanese encephalitis virus
WNV: West Nile virus
ZIKV: Zika virus
YFV: Yellow fever virus
TBEV: tick-borne Encephalitis virus
ORF: open reading frame
UTR: untranslated region
RdRp: RNA-dependent RNA polymerase
PCD: programmed cell death
PTP: permeability transition pore
MMP: mitochondrial membrane potential
IAPs: inhibitors of apoptotic protein
AIF: apoptosis-inducing factor
UPR: unfolded protein response
IRE1: inositol requiring protein-1
PERK: protein kinase RNA-like ER kinase
ATF6: activating transcription factor-6
TRAF2: TNF receptor-associated factor 2
ASK1: apoptosis signaling kinase 1
eIF2 α: eukaryotic translation initiation factor 2 α
ATF4: activating transcription factor 4
DRs: death receptors
TNF: tumor necrosis factor
DD: death domain
FADD: Fas-associated protein with death domain
DED: death effector domain
DISC: death-inducing signaling complex
tBid: truncated Bid
RIP1: receptor-interacting protein kinase
DHF: dengue hemorrhagic fever
DSS: dengue shock syndrome
ROS: reactive oxygen species
NO: nitric oxide
RIPK2: receptor-interacting serine/threonine protein kinase 2
CNS: central nervous system
RS: ribosomal stress.
